# Supplementary material for: Different promoter affinities account for specificity in MYC-dependent gene regulation
Source: eLife. 2016 Jul 27;5:e15161. doi: 10.7554/eLife.15161 (PMC4963202; doi:10.7554/eLife.15161)
Supplement: Supplementary file 3. — DOI: http://dx.doi.org/10.7554/eLife.15161.018 [file elife-15161-supp3.docx]

**Supplementary file 3**

This supplementary file lists primer sequences used in this study.

| **name** | **sequence** | **application** |
| --- | --- | --- |
| NPM1_for | TTCACCGGGAAGCATGG | ChIP-qPCR |
| NPM1_rev | CACGCGAGGTAAGTCTACG | ChIP-qPCR |
| NCL_for | CTACCACCCTCATCTGAATCC | ChIP-qPCR |
| NCL_rev | TTGTCTCGCTGGGAAAGG | ChIP-qPCR |
| HSPBAP1_for | ACCACGCAGCTTTGTTTTGA | ChIP-qPCR |
| HSPBAP1_rev | GCTAAGGTCCGGGTTAGGTA | ChIP-qPCR |
| FBXO32_for | GAGAGGATCTCAAGCGTTGC | ChIP-qPCR |
| FBXO32_rev | CTCTTCCGGCAACAAAGAGC | ChIP-qPCR |
| Ctrl_region_ch11_80MB_for | TTTTCTCACATTGCCCCTGT | ChIP-qPCR |
| Ctrl_region_ch11_80MB_rev | TCAATGCTGTACCAGGCAAA | ChIP-qPCR |
| MYC_for | CACCAGCAGCGACTCTGA | RT-qPCR |
| MYC_rev | GATCCAGACTCTGACCTTTTGC | RT-qPCR |
| CAMKV_for | TGATTTGGGACAGGTCATCA | RT-qPCR |
| CAMKV_rev | TGGAACTTCTTGCAGGTGTG | RT-qPCR |
| RGS16_for | CTGCGATACTGGGAGTACTGG | RT-qPCR |
| RGS16_rev | CCACCCCAGCACATCTTC | RT-qPCR |
| COL5A1_for | GACACCTCCAACTCCTCCAA | RT-qPCR |
| COL5A1_rev | TCTCGTCAAGGTTCCGGATC | RT-qPCR |
| ALDH3B1_for | AAGCCATCGGAGATTAGCAA | RT-qPCR |
| ALDH3B1_rev | AGCAGCTCTGGTCCACGTAT | RT-qPCR |
| B2M_for | GTGCTCGCGCTACTCTCTC | RT-qPCR |
| B2M_rev | GTCAACTTCAATGTCGGAT | RT-qPCR |
